# Activin A and ALK4 Identified as Novel Regulators of Epithelial to Mesenchymal Transition (EMT) in Human Epicardial Cells

**DOI:** 10.3389/fcell.2021.765007

**Published:** 2021-12-16

**Authors:** Esther Dronkers, Tessa van Herwaarden, Thomas J van Brakel, Gonzalo Sanchez-Duffhues, Marie-José Goumans, Anke M Smits

**Affiliations:** ^1^ Department of Cell and Chemical Biology, Leiden University Medical Center, Leiden, Netherlands; ^2^ Department of Cardiothoracic Surgery, Leiden University Medical Center, Leiden, Netherlands

**Keywords:** epicardium, EMT—epithelial to mesenchymal transition, ALK4, activin A, cardiac repair and regeneration, heart, primary cell culture

## Abstract

The epicardium, the mesothelial layer covering the heart, is a crucial cell source for cardiac development and repair. It provides cells and biochemical signals to the heart to facilitate vascularization and myocardial growth. An essential element of epicardial behavior is epicardial epithelial to mesenchymal transition (epiMT), which is the initial step for epicardial cells to become motile and invade the myocardium. To identify targets to optimize epicardium-driven repair of the heart, it is vital to understand which pathways are involved in the regulation of epiMT. Therefore, we established a cell culture model for human primary adult and fetal epiMT, which allows for parallel testing of inhibitors and stimulants of specific pathways. Using this approach, we reveal Activin A and ALK4 signaling as novel regulators of epiMT, independent of the commonly accepted EMT inducer TGFβ. Importantly, Activin A was able to induce epicardial invasion in cultured embryonic mouse hearts. Our results identify Activin A/ALK4 signaling as a modulator of epicardial plasticity which may be exploitable in cardiac regenerative medicine.

## Introduction

The epicardium, a mesothelial cell layer envelopping the heart, is increasingly recognized as a crucial contributor to heart development and repair. During cardiac development, the epicardium supplies the myocardium with cardiogenic biochemical signals and with cells such as fibroblasts, smooth muscle cells and pericytes (Dettman et al., 1998; Smits et al., 2018). Studies preventing the formation of the epicardium reported severe defects in vascularization and in myocardial compaction (Gittenberger-de Groot et al., 2000; Männer et al., 2005) demonstrating the physiological significance of the epicardium in cardiogenesis. Furthermore, disruption of epicardial behavior, for example due to genetic mutations, can contribute to congenital heart disease (Ruiz-Villalba and Pérez-Pomares, 2012).

To partake in heart development, epicardial cells undergo epithelial to mesenchymal transition (epiMT) (Dettman et al., 1998). This process is characterized by exchanging epicardial markers such as WT1, for mesenchymal proteins such as aSMA, POSTN and N-cadherin, thereby modulating their cytoskeleton and cell-cell adhesive properties. These dramatic phenotypical changes allow the cell to degrade the basal membrane and migrate into the underlying tissue.

In the healthy adult heart, the epicardium is a quiescent cell-layer. However, ischemic injury induces recapitulation of fetal epicardial processes (Zhou et al., 2011), including the upregulation of epiMT related genes (van Wijk et al., 2012). Similar to its function in development, the epicardium participates in tissue formation in the cardiac post-injury response (Duan et al., 2012; Wang et al., 2015). Importantly, it has been shown that increased epicardial activity is associated with an improved reparative response of the mammalian heart (Smart et al., 2011; Balbi et al., 2019), thereby suggesting that activation of this cell layer can be an attractive therapeutic target to improve cardiac repair. A recent finding by Mantri et al. using pseudotime analysis of chick heart single cell RNA sequencing data suggested that epiMT occurs prior to fate specification (Mantri et al., 2021), emphasizing that regulation of epiMT is an essential first step in the epicardial contribution to tissue formation. This is further substantiated by embryonal mouse models where epicardial EMT was hampered, resulting in severe defects in smooth muscle cell coverage of the vessels and in myocardial invasion (Zamora et al., 2007; Sridurongrit et al., 2008; Liu et al., 2018; Jackson-Weaver et al., 2020). Therefore, regulating epiMT is the key step to control the epicardium-driven repair response post-injury.

In order to study epiMT we have previously developed a cell culture system using human primary fetal and adult epicardial cells (Moerkamp et al., 2016). In this model, we have shown that adult epicardial cells efficiently undergo epiMT upon stimulation with Transforming growth factor (TGF)β, whereas fetal epicardial cells undergo epiMT spontaneously which is counteracted by SB431542, a TGFβ type I receptor kinase inhibitor (Moerkamp et al., 2016). TGFβ is an extensively described regulator of epithelial to mesenchymal differentiation (Kahata et al., 2018). TGFβ family ligands induce intracellular signaling responses upon activation of a transmembrane receptor complex consisting of type I and type II receptors with enzymatic serine threonine kinase activity. In the case of TGFβ1/2/3, they act by binding to TGFβ type II receptor (TGFβRII) that forms a complex with, and transphosphorylates the TGFβ type I receptor activin receptor-like kinase 5 (ALK5). Once activated, ALK5 kinase in turn phosphorylates SMAD2 and SMAD3 that partners with SMAD4 and translocations into the nucleus in order to modulate the expression of a specific subset of genes, several of them involved in EMT (Goumans and ten Dijke, 2018). However, the TGFβ pathway can also interact with other pathways, such as nuclear factor kappa B (NF-κB) (López-Rovira et al., 2000). Furthermore, the TGFβ family does not solely consist of TGFβ signaling, but also includes signaling via BMP and Activins which can signal via other type I and type II receptor complexes. The role of these related pathways in epiMT is yet unexplored (Dronkers et al., 2020).

In order to study epiMT regulating pathways in more detail, we have further exploited our primary cell culture system. The unique feature of having both highly active fetal epicardial cells and inducible adult cells allows for studying pathway inhibitors and stimulants of epiMT. When interrogating this model, we identified Activin A and its receptor ALK4 signaling as novel regulators of epicardial plasticity *in vitro*. Additionally we show that Activin A can indeed induce epicardial invasion in *ex vivo* cultured mouse hearts.

## Methods

### Collection of Human Cardiac Tissue

Human adult heart auricles were collected anonymously as surgical waste from patients undergoing cardiac surgery under general informed consent. Human fetal cardiac tissue was collected with informed consent and anonymously from elective abortion material of fetuses with a gestational age between 10 and 20 weeks. This research was carried out according to the official guidelines of the Leiden University Medical Center and approved by the local Medical Ethics Committee (number P08.087). This research conforms to the Declaration of Helsinki.

### Cell Culture and Experiments

#### Cell Culture

Epicardial tissue was isolated as described (Dronkers et al., 2018). Briefly, the epicardial layer was stripped from the cardiac tissue and minced followed by several rounds of incubation with 0.25% trypsin/EDTA. The suspension was passed through a syringe and filtered to obtain a single cell suspension. Cells were seeded on 0.1% gelatin (Sigma Aldrich) coated plates and cultured in EPDC medium which consists of Dulbecco’s modified Eagle’s medium (DMEM low- glucose, Gibco) and Medium 199 (M199, Gibco) mixed in a 1:1 ratio, supplemented with 10% fetal bovine serum (heat inactivated for 25 min at 56°C, Biowest), and 100 U/mL penicillin (Roth) and 100 mg/ml streptomycin (Roth). Cells were cultured in the presence of 10 µM SB431542 (Tocris) at 37°C in 5% CO_2_.

#### Ligand and Inhibitor Stimulation

To reduce patient variation, only those cell isolations were included that upon isolation displayed a clear epithelial phenotype shown by a cobblestone morphology. For experiments, fetal or adult EPDCs were trypsinized and seeded in a ∼30–50% confluency. The next day, cells were stimulated for five days with either TGFβ3 (1 ng/ml, R and D systems), BMP6 (50 ng/ml, Peprotech), TNFα (10 ng/ml, Peprotech), Activin A (50 ng/ml, Peprotech), SB431542 (10 μM, Tocris), LY2157299 (20 μM, Calbiochem), TGFβ1/2/3 monoclonal capture antibody (1D11) (1 μg/ml, ThermoFisher), control capture antibody (13C4) (1 μg/ml, Genzyme), LDN212854 (100 nM, Axon Medchem), Bay 11-7085 (5 μM, Sigma-Aldrich) or Follistatin 288 (5 μg/ml, homemade). Working concentrations of ligands and inhibitors were determined based on concentrations series experiments, selecting the lowest concentration that exerted epiMT. For the factors that did not elicit an effect, we used the concentration that is commonly described in literature.

#### Staining and Imaging

Cells were fixed in 4% PFA, washed and blocked in 1%BSA/0.1% Tween 20/PBS and incubated with a primary antibody against human αSMA (Human alpha-Smooth Muscle Actin Alexa Fluor^®^ 488-conjugated Antibody, R&D systems), HA (12CA5, Roche) or ALK4 (Activin A Receptor Type IB/ALK-4, Abcam). Then, cells were incubated with a secondary antibody (Alexa Fluor 488, 555 or 647, Thermo Scientific) combined with phalloidin conjugated antibody (Rhodamine Phalloidin, Invitrogen). Lastly, cells were stained with DAPI (Thermo Scientific). Imaging was performed using the Leica AF6000.

#### Isolation of mRNA and qPCR

mRNA was isolated using ReliaPrep™ RNA Miniprep Systems (Promega). The mRNA concentration and purity were measured using NanoDrop 1000 Spectrophotometer (Thermo Fisher Scientific) followed by cDNA synthesis using the RevertAid H Minus First Strand cDNA Synthesis Kit (Thermo Fisher Scientific). qPCR was performed in a 384 wells format using SYBR Green (Promega) and run on a CFX384 Touch™ Real-Time PCR Detection System (Bio-Rad). Expression levels were normalized for two reference genes (*HPRT1* and *TBP*) which were designed and tested for robust expression in adult and fetal EPDCs and in epithelial and mesenchymal samples using geNorm (VandeSompele 2002). Primers sequences are provided in Supplementary Table S1.

#### Adenoviral Transduction

For transduction with constitutively active ALK4 (Ad-caALK4), or wild type ALK4 (Ad-ALK4-OE), adenovirus was generated as described (Fujii et al., 1999), and produced using ViraPower adenoviral expression system (Life technologies). To determine the effect on cellular phenotype and epiMT markers, adult EPDCs were transduced with Ad-caALK4, Ad-ALK4-OE or control Ad-LacZ virus for 24 h and subsequently cultured for four days. To establish expression of EMT transcription factors, mRNA of adult EPDCs was isolated 20 h after transduction.

#### Baseline Gene Expression Profiles

To eliminate the potential effect of SB431542 on baseline levels, it was first established that the effect of ALK4/5/7 kinase inhibition expired 3 h after removal of SB. Therefore, adult and fetal cobble EPDCs with a confluency of 60–80% were cultured for 3 h in the absence of SB431542 whereafter RNA was isolated.

### *Ex vivo* Invasion Assay

All animal experiments were performed according to protocols approved by the animal welfare committee of the Leiden University Medical Center and conform the guidelines from Directive 2010/63/EU of the European Parliament on the protection of animals used for scientific purposes. Female Rosa26^mTmG/mTmG^ mice were set-up for timed matings with male Wt1^creERT2/+^. The presence of a plug in the morning was denoted as embryonic day (E)0.5. At E9.5, the mother was injected with tamoxifen (2 mg) to label the pro-epicardial cells. After three days, when the epicardium has covered the heart, embryos were isolated from which the embryonic heart was dissected and cultured based on a previously published protocol (Jackson-Weaver et al., 2020). Embryonic tissue was cultured in DMEM high glucose and M199 mixed in a 1:1 ratio supplemented with 0.1% FBS and 100 U/mL penicillin and 100 mg/ml streptomycin at 37°C in 5% CO_2_. After 24 h, the culture medium of the embryos carrying the Cre + genotype was supplemented with 2 ng/ml FGF with or without 200 ng/ml Activin A and incubated for another 48 h. The tissue was fixed in 4% PFA, embedded in paraffin, sectioned, and stained as described (Kruithof et al., 2020) using the following antibodies: α-GFP(Abcam, ab13970), α-Tropomyosin (Sigma-Aldrich, T9283) and α-Wt1 (Abcam, ab89901).

### Quantification and Statistics

αSMA surface area was quantified by taking four blinded pictures per condition per cell isolation, which subsequently were analyzed in an unbiased fashion using Fiji software and corrected for the number of DAPI + nuclei per picture. For every experiment, the n number is indicated, referring to the number of individual cell isolations that have been used. Displayed pictures are representative for multiple observations. Statistics were performed using Graphpad 9.0.1 software. Only relevant comparisons, which are indicated in the figures by a stripe, were statistically tested. For every experiment, the performed statistical test is indicated in the figure legend. Significance was considered when *p* < 0.05.

## Results

To study EMT in epicardial cells (epiMT), we established a model consisting of primary epicardial derived cells (EPDCs) isolated from human adult and fetal cardiac specimens. In agreement with our previous results, EPDCs cultured in the presence of the ALK4/5/7 kinase inhibitor SB431542 (SB) maintained an epithelial phenotype characterized by a round cobblestone morphology (Supplementary Figures S1A,B, orange arrows) (Moerkamp et al., 2016). After exposure to exogenous TGFβ, EPDCs underwent epiMT, demonstrated by a change towards a spindle-shaped, mesenchymal cell morphology (Supplementary Figures S1A,B, bright field). This was accompanied by a high expression α Smooth Muscle Actin (αSMA).

**FIGURE 1:**
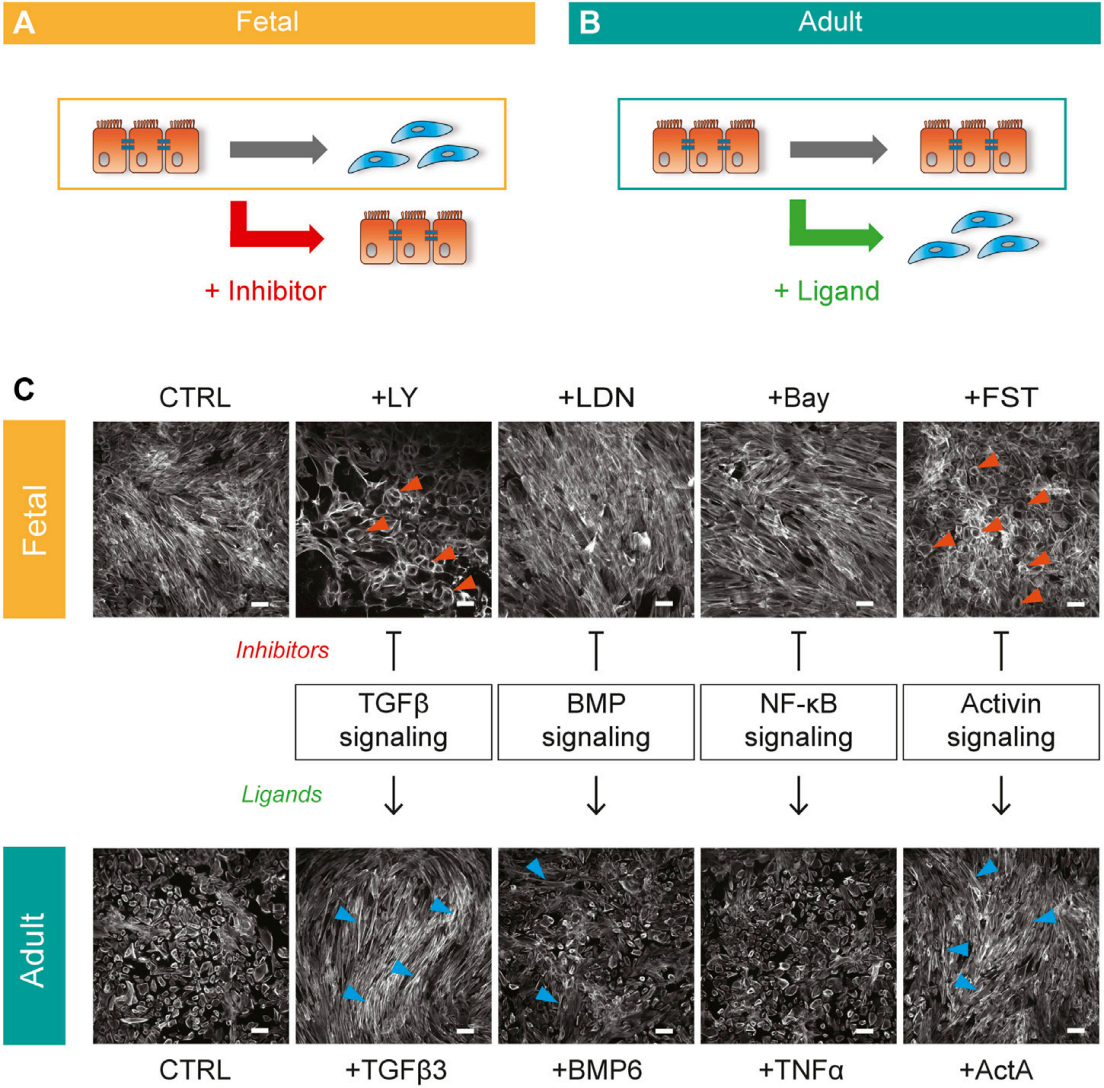
Bi-directional cell culture model to unravel epiMT regulating pathways **(A)** Schematic representation of spontaneous fetal epiMT as a platform to identify inhibitors of epiMT. **(B)** Schematic representation of adult EPDCs as a model to identify inducers of epiMT. **(C)** Combined bi-directional model of human fetal **(top)** and adult **(bottom)** EPDCs cultured for 5 days with multiple inhibitor-ligand combinations to assess pathway involvement in epiMT. Phalloidin staining shows the epithelial (orange arrows) and mesenchymal (blue arrows) phenotype. Inhibitors: LY-2157299 (LY), LDN212854 (LDN), Bay 11-7085 (Bay), Follistatin (FST), Ligands: Transforming growth factor beta 3 (TGFβ 3), bone morphogenetic protein 6 (BMP6), tumor necrosis factor alpha (TNFα), Activin A (ActA). Representative for *n* = 3. Scale bar: 100 µM.

Importantly, removing SB from the culture medium (CTRL) did not affect the morphology of adult EPDCs (Supplementary Figure S1B), while in fetal EPDCs the absence of SB was sufficient to initiate epiMT (Supplementary Figure S1A). The ability to spontaneously undergo epiMT makes the human fetal cell culture system an attractive model to identify inhibitors of epiMT (Figure 1A). Conversely, adult EPDC can be used to detect inducers of epiMT (Figure 1B). Therefore, to identify novel pathways involved in human epiMT, we applied our *in vitro* model as a bi-directional cell culture system, allowing parallel analysis of pathway inhibitors in fetal EPDCs and their affiliated ligands in adult EPDCs. The fact that SB blocks spontaneous epiMT in fetal cells suggests that this process is governed via an ALK4/5/7 mediated pathway. First, we confirmed the role of TGFβ signaling in epiMT using an alternative ALK4/5/7 kinase inhibitor LY2157299 (LY). While TGFβ induced an obvious morphological change compared to adult epicardial control conditions -as shown by the appearance of F-Actin stress fibers (Figure 1C, blue arrows)- LY prevented spontaneous fetal epiMT. Cells maintained a cobblestone morphology, with a cortical organization of F-Actin fibers as revealed by phalloidin staining at the inner cell surface (Figure 1C, orange arrows), which was distinctly different from the fetal control condition, thereby confirming our previous finding. Next, we explored TGFβ-related signaling pathways which have been associated with EMT, namely BMP signaling (Dituri et al., 2019) and nuclear factor kappa B (NF-κB) signaling (López-Rovira et al., 2000). After stimulation with BMP6, adult EPDCs displayed some patches of spindle shaped cells, but no gross morphological switch was observed compared to control cells. Moreover, since the BMP type I receptor kinase inhibitor LDN-212854 (LDN) was unable to block epiMT we disregarded BMP signaling as a major player in this process. Modulation of the NF-κB pathway did not morphologically alter either the fetal or the adult epicardial cells. However, exogenous Activin A stimulation of adult EPDCs led to a clear transition towards a spindle shaped morphology (Figure 1C, blue arrows). Moreover, fetal EPDCs incubated with the Activin natural antagonist Follistatin (FST) maintained an epithelial phenotype (Figure 1C, orange arrows). The combination of these two observations points towards a role for a previously unknown ability of Activin A signaling to regulate epiMT.

**FIGURE 2:**
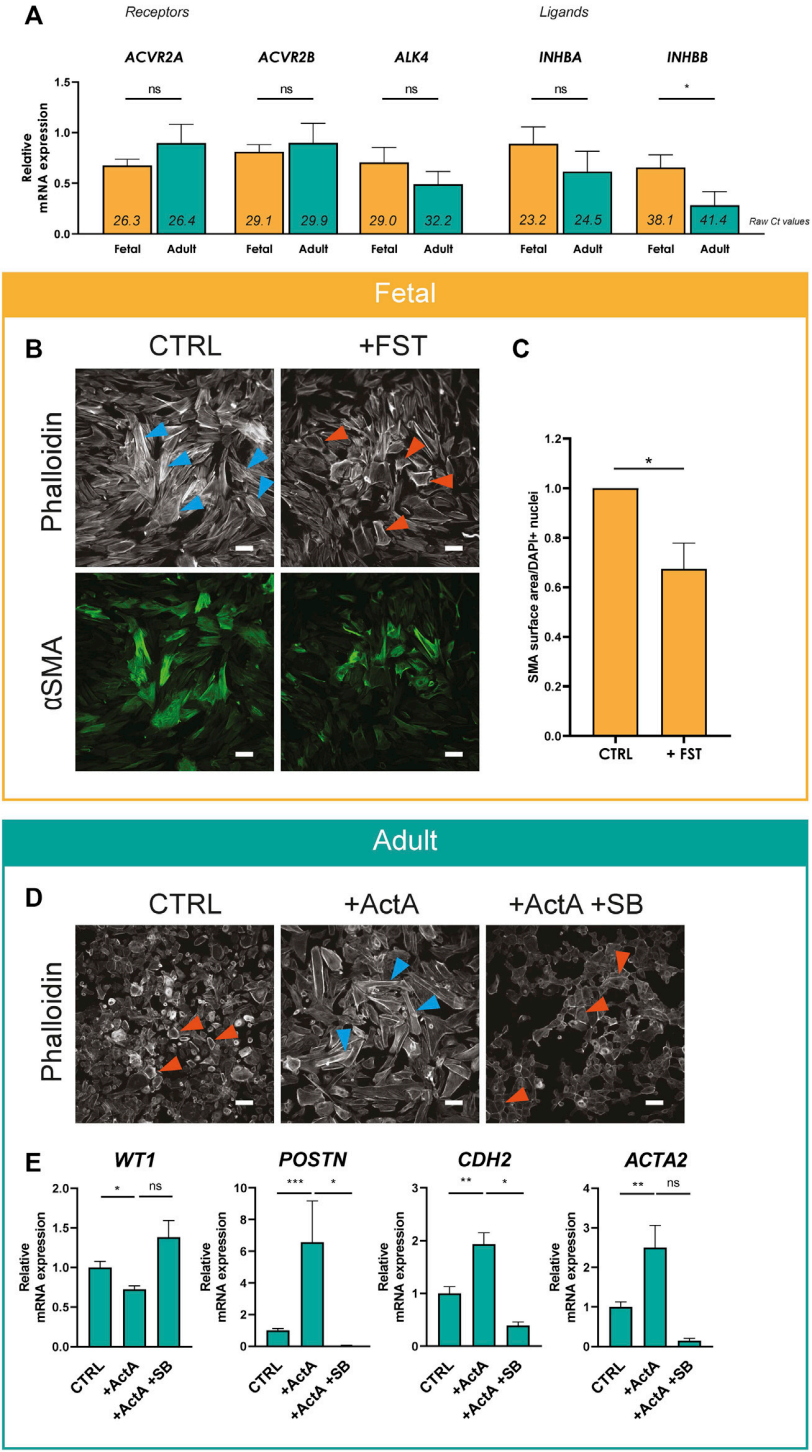
Activin A signaling regulates epiMT **(A)** mRNA expression levels for Activin receptor type 2A (*ACVR2A*), Activin receptor type 2B (*ACVR2B*), Activin receptor type 1B (*ALK4*), Inhibin subunit beta A (*INHBA*) and Inhibin subunit beta B (*INHBB*) determined in cobble fetal and adult EPDCs cultured for 3 h after removal of SB (*n* = 6). Raw Ct values per condition are shown in italics. * = *p* < 0.05, ns = not significant (unpaired Student’s *t*-test). Data are displayed as mean + SEM. **(B)** Immunofluorescent staining for phalloidin and αSMA in fetal EPDCs cultured for 5 days in control (CTRL) or FST containing medium. Orange arrows indicate examples of epithelial cobblestone-shaped cells, blue arrows of mesenchymal spindle-shaped cells. Scale bar: 100 µm. **(C)** Quantification of αSMA positive surface area of FST treated cells relative to CTRL (*n* = 7). * = *p* < 0.05 (paired Student’s T-Test). **(D)** Phalloidin staining in adult EPDCs cultured for 5 days in control medium (CTRL), medium containing Activin A, or Activin A in combination with SB431542. Scale bar: 100 µm. **(E)** mRNA expression levels of *WT1*, *POSTN*, *CDH2* and *ACTA2* of adult EPDCs cultured for 5 days in the presence of ActA (*n* = 7) or ActA + SB (*n* = 3), relative to CTRL. * = *p* < 0.05, ** = *p* < 0.01, ns = not significant (mixed-effects analysis, Sidak’s multiple comparisons test).

We continued to explore the role of Activin A signaling in epiMT. First, we determined the expression levels of relevant signaling components related to Activin. Activin homo- or heterodimeric ligands are composed by combinations of two subunits encoded by *INHBA* and/or *INHBB,* which signal by binding to the type II receptors ACVR2A or ACR2B, and type I receptor ALK4. The presence of all components could be established in both adult and fetal EPDCs (Figure 2A, raw Ct values in italics indicate presence of mRNA transcripts). Furthermore, ALK4, INHBA and INHBB mRNA showed a trend towards higher expression in fetal EPDCs.

Next, we validated the effect of Activin A signaling on epiMT markers in more detail. Fetal EPDCs treated with FST displayed a significant reduction of αSMA expression compared to control cells, confirming the prevention of epiMT (Figures 2B,C and Supplementary Figure S2 for DAPI). In adult EPDCs, the Activin A-induced phenotypical change towards a mesenchymal cell type was accompanied by a significant decrease of epicardial marker WT1, and an increase of mRNA expression of mesenchymal markers *ACTA2* (encoding αSMA), *POSTN* and *CDH2* confirming the occurrence of epiMT (Figures 2D,E). However, this did not result in a large change in αSMA protein levels (Supplementary Figure S3).

To study the Activin A signaling pathway in more detail, we focused on the type I receptor ALK4. Noteworthy, besides inhibition of ALK5 kinase activity, SB and LY also inhibit the Activin type I receptor kinase ALK4. As expected based on its signaling via ALK4, Activin A initiated epiMT could be blocked by SB (Figures 2D,E).

**FIGURE 3:**
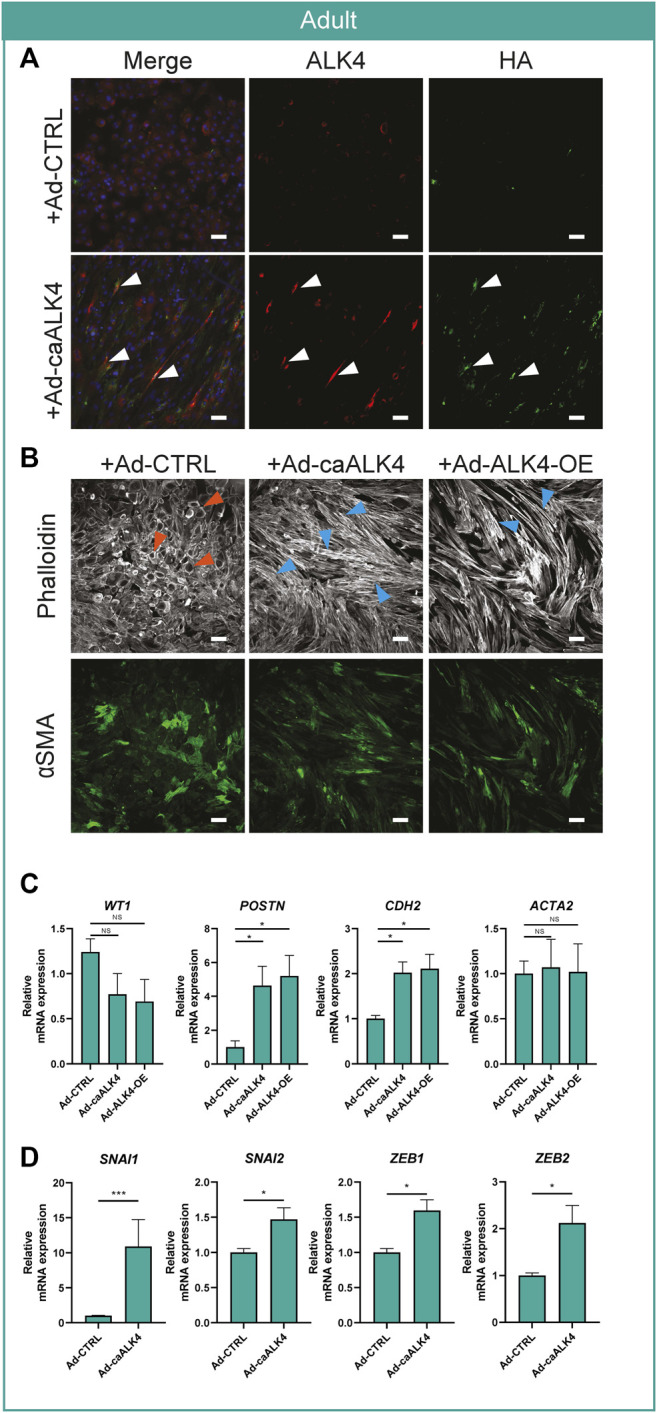
Activin receptor ALK4 overexpression induces epiMT in adult EPDCs **(A)** Representative example of ALK4 and HA staining in Ad-CTRL and Ad-caALK4 transduced adult EPDCs. Arrows indicate co-localization of ALK4 and HA in Ad-caALK4 treated cells. **(B)** Phalloidin and αSMA staining of adult EPDCs transduced with Ad-CTRL, Ad-caALK4 or Ad-ALK4-OE (*n* = 3). Scale bar: 100 µm. Orange arrows indicate examples of epithelial cobblestone-shaped cells, blue arrows of mesenchymal spindle-shaped cells. **(C)** mRNA expression levels of *WT1*, *POSTN*, *CDH2* and *ACTA2* (*n* = 3) of adult EPDCs cultured for 5 days after transduction with Ad-caALK4 or Ad-ALK4-OE, relative to Ad-CTRL. * = *p* < 0.05, ns = not significant (mixed-effects analysis, Sidak’s multiple comparisons test). **(D)** mRNA expression levels of EMT transcription factors *SNAI1, SNAI2, ZEB1, ZEB2* (*n* = 3) of adult EPDCs cultured for 20 h after transduction with Ad-caALK4 or Ad-ALK4-OE, relative to Ad-CTRL. * = *p* < 0.05, ** = *p* < 0.01, *** = *p* < 0.001, ns = not significant (mixed-effects analysis, Sidak’s multiple comparisons test).

To further confirm that ALK4 signaling is relevant for epiMT, adult EPDCs were transduced with an adenovirus expressing constitutively active ALK4 (Ad-caALK4) bound to an HA-tag. Successful viral transduction was confirmed by HA-tag protein expression (Supplementary Figure S4), and co-localisation of HA and ALK4 protein (Figure 3A). Within five days, Ad-caALK4 transduced adult EPDCs robustly displayed a mesenchymal phenotype, and an increased expression of POSTN and N-cadherin (Figures 3B,C). Furthermore, Ad-caALK4 transduction elicited an extensive upregulation of EMT transcription factors (Figure 3D). In addition, adenoviral overexpression of wild type ALK4 in adult EPDCs (Ad-ALK4-OE) provoked a quick and profound induction of epiMT (Figures 3B,C), which could suggest that ALK4 receptor availability impedes adult EPDCs to undergo epiMT *in vitro*.

**FIGURE 4:**
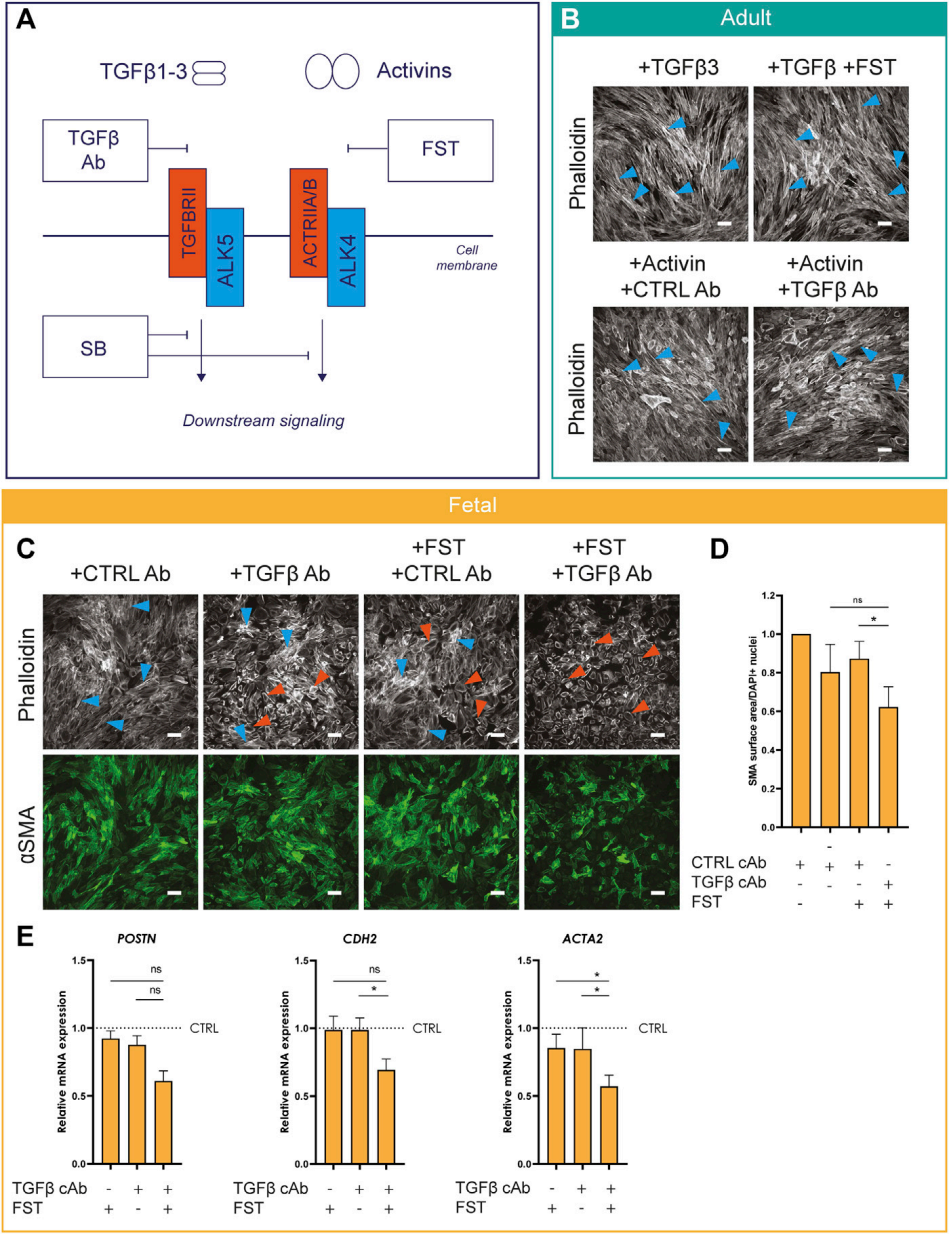
Activin A-induced epiMT is independent of TGFβ signaling **(A)** Schematic overview of TGFβ and Activin signaling and their concomitant inhibitors.**(B)** Phalloidin staining of adult EPDCs cultured for 5 days in the presence of TGFβ3, TGFβ3+FST, ActA + CTRL capture Ab (cAb), or Activin A + TGFβ cAb (*n* = 3). Scale bar: 100 µm. **(C)** Phalloidin and αSMA staining (*n* = 5) of fetal EPDCs cultured for 5 days in the presence of Activin A or TGFβ inhibitors. Scale bar: 100 µm. Orange arrows indicate examples of epithelial cobblestone-shaped cells, blue arrows of mesenchymal spindle-shaped cells. **(D)** Quantification of αSMA area (*n* = 4), * = *p*< 0.05 (mixed-effects analysis, Sidak’s multiple comparisons test). Data are displayed as mean +SEM. **(E)** mRNA expression levels of *POSTN*, *ACTA2*, *CDH2* (*n* = 4) of fetal EPDCs cultured for 5 days. * = *p* < 0.05, ns = not significant (mixed-effects analysis, Sidak’s multiple comparisons test). Data are displayed as mean + SEM.

Thus far, we established that Activin A and ALK4 can regulate epiMT *in vitro*. Next, we explored potential synergistic effects between TGFβ and Activins by stimulating with one ligand and simultaneously blocking the alternate ligand-receptor interaction with a ligand neutralizing antibody, as schematically depicted in Figure 4A. As such, adult EPDCs were treated with TGFβ in combination with FST, or Activin A in combination with a TGFβ capture antibody (cAb) (for cAb effectivity tests, see Supplementary Figures S5,S6). EpiMT induction by TGFβ could not be blocked by the Activin inhibitor FST. Likewise, Activin A-induced epiMT was not prevented by the TGFβ cAb (Figure 4B and Supplementary Figures S5, S6 for controls). This suggests that both ALK5 and ALK4 mediated signaling independently have the ability to induce epiMT. Next, we assessed the effect of combined TGFβ and Activin blockade on fetal epicardial cells. Importantly, combined treatment with TGFβ cAb and FST exhibited an additive effect compared to FST treatment alone in fetal cells, as assessed by αSMA protein expression levels, and on mRNA levels of mesenchymal genes (Figures 4C–E and Supplementary Figure S7 for DAPI). Taken together, our results demonstrate that Activin A and TGFβ can drive epiMT independently.

**FIGURE 5:**
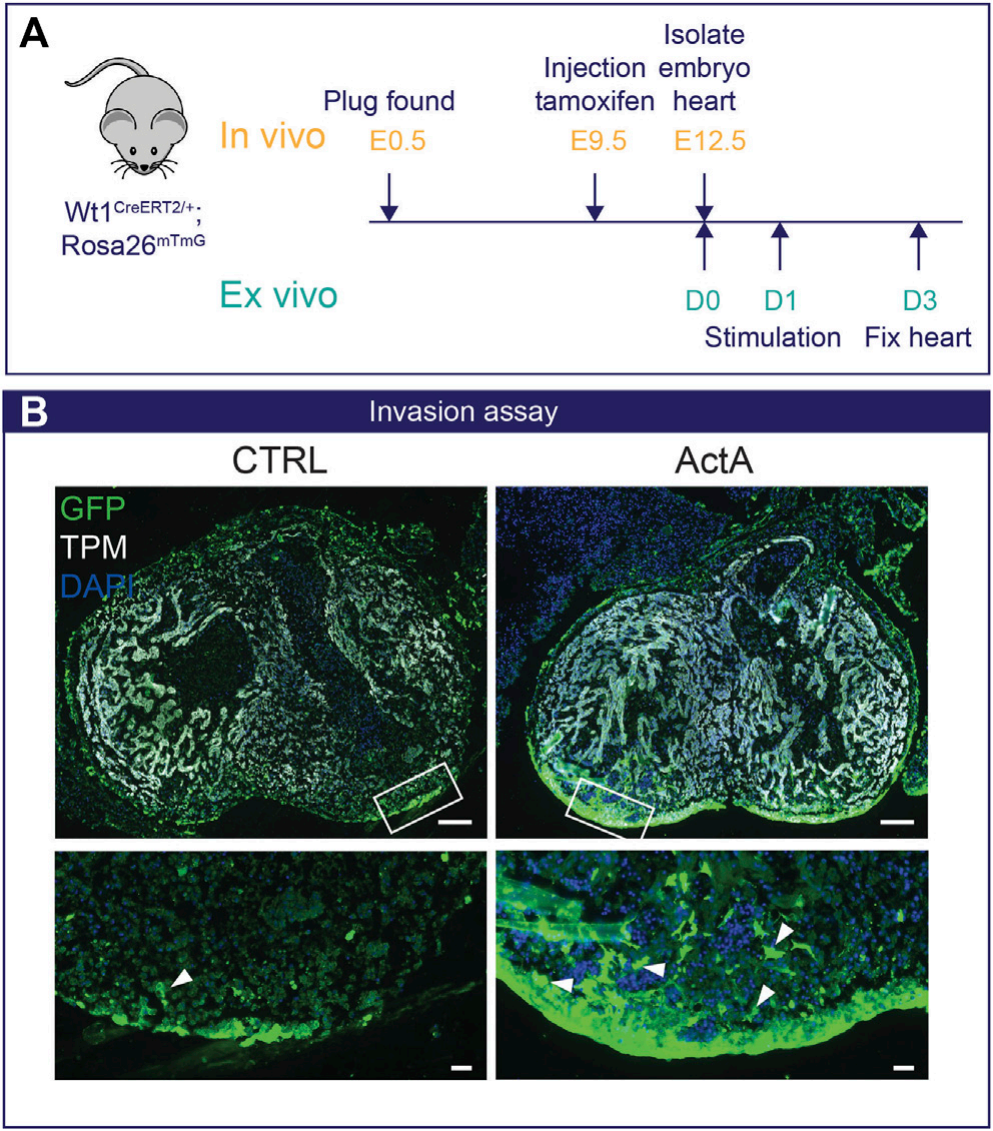
Activin A signaling initiates epicardial invasion in mouse heart **(A)** Experimental design: Pregnant dams are injected with tamoxifen at E9.5 and embryos are isolated on E12.5. Wt1^creERT2/+^;Rosa26^*mTmG*/+^ embryos were cultured *ex vivo* for 24 h and then stimulated with either 200 ng/ml Activin A (*n* = 3) or control medium for 48 h.**(B)** Immunofluorescent staining for GFP (green), tropomyosin (TPM, white), and DAPI (blue). White arrows indicate Wt1-Cre-GFP+ epicardial cells that have invaded the myocardium. Scale bar: 100 µm for overview and 20 µm for zoom-in.

Finally, we validated our *in vitro* findings in a physiologically relevant setting of *ex vivo* murine embryonic heart cultures with an epicardial specific lineage trace system to study epicardial invasion (Figure 5A). Wt1^creERT2/+^ Rosa26^*mTmG*^ embryos were exposed *in utero* to tamoxifen at embryonic day E (9.5) to label Wt1^+^ epicardial cells with GFP. At E12.5, hearts were isolated and cultured *ex vivo* as depicted in Figure 5, and invasion of epicardial cells into the myocardium was analyzed by fluorescent microscopy. Under control conditions, GFP^+^ epicardial cells remained mostly at the surface of the heart. Interestingly, stimulation with Activin A induced a profound induction of epicardial invasion (Figure 5B), confirming that the Activin pathway is relevant for epiMT in a whole organ setting.

## Discussion

The external layer of the heart, so called epicardium, has been implicated in key developmental processes and regenerative episodes. However, approaches to regulate epicardial plasticity remain elusive. Using a unique screening model consisting of human adult and fetal EPDCs, here we demonstrate that 1) epiMT can be regulated by Activin A (ActA) and ALK4 receptor activation, which 2) occurs in a TGFβ-signaling independent manner. As validation of these findings, we showed that 3) Activin A can initiate epicardial invasion in *ex vivo* heart tissue.

We have previously shown that primary fetal epicardial cells display an augmented epithelial-mesenchymal plasticity and readily undergo epiMT, while adult epicardial cells are relatively quiescent and only undergo epiMT when stimulated (Moerkamp et al., 2016). Combining these two models into a bidirectional cell culture system revealed Activin signaling as a novel regulator of epiMT. A role for Activin A and its receptor ALK4 in epicardial cells has not been described to date, but this signaling pathway is known to be able to promote EMT in multiple cancer cell lines (Valcourt et al., 2005; Murakami et al., 2010; Basu et al., 2015; Bauer et al., 2015; Dean et al., 2017). In our study, we established epiMT based on morphological changes, gene expression profiles of both EMT transcription factors and epicardial and mesenchymal markers, F-actin localization, αSMA protein expression, and invasion capacity, as recommended by Yang et al, (2020). Interestingly, we observed a trend towards higher expression of ALK4, INHBA, and INHBB mRNA in fetal compared to adult EPDCs. Moreover, increasing ALK4 receptor availability on the surface of adult epicardial cells using adenoviral overexpression was sufficient to induce spontaneous epiMT in adult cells. Combined with the observation that fetal epiMT can partially be prevented by removal of Activin ligand with FST suggests a higher sensitivity of fetal EPDCs for Activin signaling, which may be one of the reasons why these cells are more prone to undergo EMT compared to adult EPDCs.

We demonstrated that both TGFβ and Activin A induce human epiMT independently. Interestingly, although TGFβ has been recognized as a central regulator of epiMT (Dronkers et al., 2020), the TGFβ cAb by itself was not able to prevent spontaneous epiMT in fetal epicardial cells. This suggests that other factors, such as Activin A, may compensate for the inactivation of TGFβ signaling. Activin A and ALK4 appear to induce less SMA expression compared to TGFβ which suggest that Activin signaling follows a separate differentiation path. In addition, our observation that incubation with recombinant FST in addition to the TGFβ cAb, prevents spontaneous epiMT also points at Activin A/ALK4 signaling as an independent regulator of epiMT. The same principle has been shown in colon cancer cells, where the joint effect of TGFβ and Activin was vital for pro-metastatic function (Staudacher et al., 2017), which is related to EMT and invasion. In hindsight, the shared effects of TGFβ/ALK5 and Activin A/ALK4 signaling might have been overlooked in other studies since SB is often regarded as an ALK5 kinase inhibitor, while it actually targets ALK4, 5 and, 7 activity. Therefore, while the importance of TGFβ in epiMT has been established multiple times using SB, this approach likely masked the involvement of Activin signaling via ALK4.

To confirm our *in vitro* findings, we took advantage of an *ex vivo* cultured embryonic mouse heart model. In this system, Activin A endows the cells with more migratory and invasion properties, suggesting the presence of the ALK4 receptor and implicating that Activin A signaling could be of importance in cardiac development and regeneration. The necessity of epicardial Activin signaling during the development of the heart has not been studied, mainly because most of the Activin-related KO mice do not show a cardiac phenotype (Namwanje and Brown, 2016) or die at an early developmental stage before heart formation is initiated. However, the availability of Activin A in (sub)epicardial tissue has been reported (Feijen et al., 1994; Lupu et al., 2020), and therefore the secreted protein should be able to reach epicardial cells and initiate signaling. The presence of ALK4 is difficult to assess because most antibodies are not suitable for immunostainings. Nevertheless, a single cell RNA sequencing dataset of embryonic mouse epicardium indicates mRNA expression of ALK4 in a subset of epicardial cells (Lupu et al., 2020).

To conclude, with this study we add a novel pathway to epiMT regulation. As the epicardium has been proposed as an endogenous source for increased repair of injured cardiac tissue, our findings can serve as a starting point for further investigation into the therapeutic role of epicardial Activin A and ALK4 signaling in development and cardiac injury.

## Data Availability

The raw data supporting the conclusions of this article will be made available by the authors on request, without undue reservation.
