# Supplementary figures and images for: Activin A and ALK4 Identified as Novel Regulators of Epithelial to Mesenchymal Transition (EMT) in Human Epicardial Cells
Source: Front Cell Dev Biol. 2021 Dec 16;9:765007. doi: 10.3389/fcell.2021.765007 (PMC8716764; doi:10.3389/fcell.2021.765007)

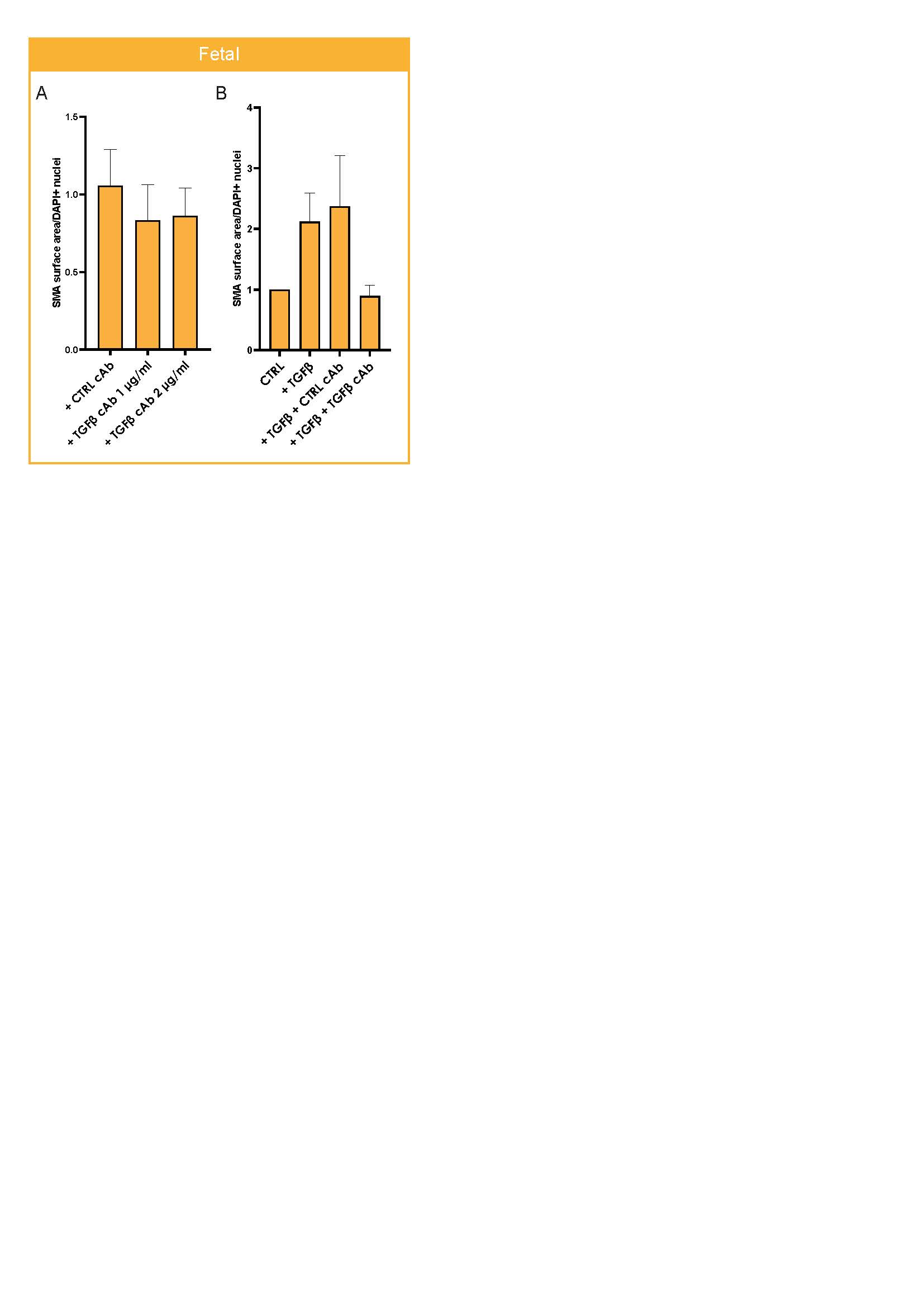

Supplement: Supplementary file 2 [file Image6.jpg]

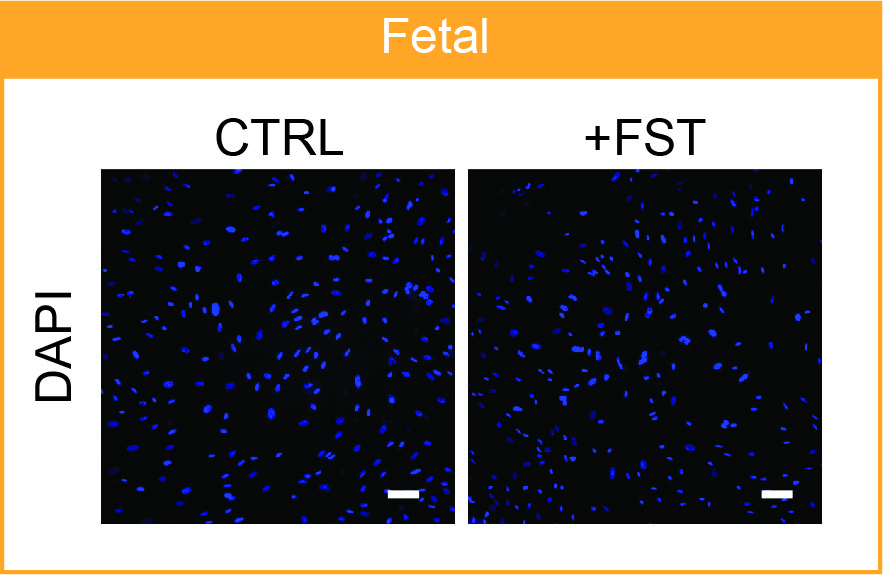

Supplement: Supplementary file 4 [file Image2.jpg]

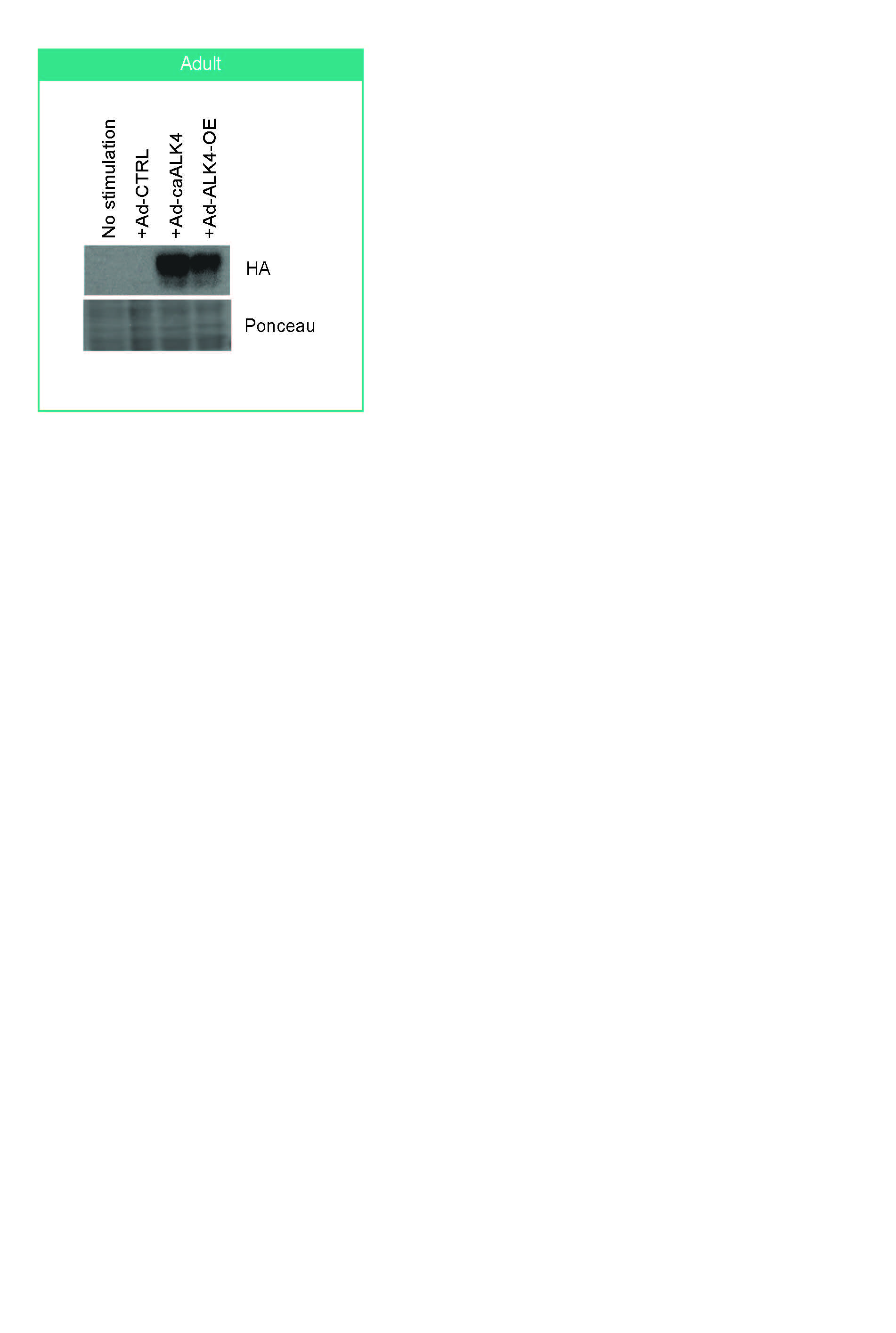

Supplement: Supplementary file 6 [file Image4.jpg]
