# Supplementary material for: Activin A and ALK4 Identified as Novel Regulators of Epithelial to Mesenchymal Transition (EMT) in Human Epicardial Cells
Source: Front Cell Dev Biol. 2021 Dec 16;9:765007. doi: 10.3389/fcell.2021.765007 (PMC8716764; doi:10.3389/fcell.2021.765007)
Supplement: Supplementary file 8 [file DataSheet1.docx]

Supplemental figure legends

**Fig. S1 | EpiMT in adult and fetal EPDCs**

Representative brightfield pictures and phalloidin and αSMA immunostaining of human fetal (A), and adult (B) epicardial derived cells (EPDCs) cultured for 5 days in the presence of SB431542 (SB), empty culture medium (CTRL) or TGFβ3 (representative images for n=5). Orange arrows indicate examples of epithelial cobblestone-shaped cells, blue arrows of mesenchymal spindle-shaped cells. Scale bar: 100 µM.

**Fig. S2 | DAPI staining of CTRL and FST treated fetal EPDCs.**
DAPI staining corresponding to images in Fig.2B. Scale bar: 100 µm.
**Fig. S3 | ActA does not enhance αSMA protein expression in adult EPDCs.**

αSMA and DAPI staining corresponding to images in Fig.2D complemented with αSMA and DAPI staining of TGFβ3 treated cells from the same experiment. Scale bar: 100 µm.
**Fig. S4 | Western blot confirms viral transduction of Ad-caALK4 in adult EPDCs**

Representative example of western blot of HA-tag presence in adult EPDCs stimulated with empty culture medium (CTRL), adenoviral LacZ (Ad-CTRL), adenoviral constitutively active ALK4 (Ad-caALK4) or adenoviral overexpression of wild type ALK4 (Ad-ALK4-OE) (n=2). Presence of protein in all lanes was confirmed using ponceau staining.

**Fig. S5 | Effectivity of TGFβ and Activin signaling inhibitors.**

Phalloidin staining of adult EPDCs cultured for 5 days in the presence of ligand and inhibitor to proof effectivity of inhibitors. Scale bar: 100 µm.
**Fig. S6 | Effectivity of TGFβ cAb**

(A) Five-day treatment of fetal EPDCs with TGFβ cAb shows that 1 µg/mL is sufficient to block TGFβ as a higher concentration does not enhance the effect (n=5). (B) Five-day treatment with TGFβ cAb can completely block the SMA-induction of exogenous TGFβ in fetal EPDCs, demonstrating its effectivity (n=4).

**Fig. S7 | DAPI staining of TGFβ cAb and FST** **treated fetal EPDCs.**
DAPI staining corresponding to images in Fig.4C. Scale bar: 100 µm.

**Supplemental table 1 | Primer sequences for qPCR**

|  | Forward | Reverse |
| --- | --- | --- |
| *ALK4* | GCTCGAAGATGCAATTCTGG | TTGGCATACCAACACTCTCG |
| *ACVR2A* | GCATCACAAGATGGCCTACC | CCAGGCAAACTGTAGACTTC |
| *ACVR2B* | ATGTGGACATCCATGAGGAC | TGAAGATCTCCCGTTCACTC |
| *INHBA* | ATAGCCCCTTTGCCAACCTC | GCTGGGCAACTCTATGAGC |
| *INHBB* | ATGATTGTGGAGGAGTGCGG | TGTCAGTTGAGGGTTCTCGC |
| *WT1* | CAG CTT GAA TGC ATG ACC TG | TAT TCT GTA TTG GGC TCC GC |
| *POSTN* | GGAGGCAAACAGCTCAGAGT | GGCTGAGGAAGGTGCTAAAG |
| *CDH2* | CAGACCGACCCAAACAGCAAC | GCAGCAACAGTAAGGACAAACATC |
| *ACTA2* | CCGGGAGAAAATGACTCAAA | GAAGGAATAGCCACGCTCAG |
| *SNAI1* | CCAGTGCCTCGACCACTATG | CTGCTGGAAGGTAAACTCTGGA |
| *SNAI2* | CGGACCCACACATTACCTTGT | TTCTCCCCCGTGTGAGTTCTA |
| *ZEB1* | AGCAGTGAAAGAGAAGGGAATGC | GGTCCTCTTCAGGTGCCTCA |
| *ZEB2* | CAC AAG CCA GGG ACA GAT CA | TTG CCA CAC TCT GTG CAT TTG |
| *HPRT1* | CTCATGGACTGATTATGGACAGGAC | GCAGGTCAGCAAAGAACTTATAGCC |
| *TBP* | TGGAAAAGTTGTATTAACAGGTGCT | GCAAGGGTACATGAGAGCCA |
